# A possible unique ecosystem in the endoglacial hypersaline brines in Antarctica

**DOI:** 10.1038/s41598-022-27219-2

**Published:** 2023-01-05

**Authors:** M. Guglielmin, M. Azzaro, P. Buzzini, D. Battistel, M. Roman, S. Ponti, B. Turchetti, C. Sannino, L. Borruso, M. Papale, A. Lo Giudice

**Affiliations:** 1 Department of Theoretical and Applied Sciences, Insubria University, Via Dunant, 3, 21100 Varese, Italy; 2 Institute of Polar Sciences, National Research Council, Spianata S. Raineri. 86, 98122 Messina, Italy; 3 Department of Agricultural, Food and Environmental Sciences, University of Perugia, Borgo XX Giugno 74, 06121 Perugia, Italy; 4 Department of Environmental Sciences, Informatics and Statistics, University Ca’ Foscari of Venice, Via Torino, 155, 30172 Mestre, VE Italy; 5 Faculty of Science and Technology, Free University of Bozen-Bolzano, Piazza Università 5, 9100 Bozen-Bolzano, Italy; 6 Climate Change Research Center, Insubria University, Via Regina Teodolinda, 37, 22100 Como, Italy

**Keywords:** Microbiology, Biogeochemistry, Ecology, Environmental sciences, Planetary science

## Abstract

Here, we present the results related to a new unique terrestrial ecosystem found in an englacial hypersaline brine found in Northern Victoria Land (Antarctica). Both the geochemistry and microbial (prokaryotic and fungal) diversity revealed an unicity with respect to all the other known Antarctic brines and suggested a probable ancient origin mainly due a progressive cryoconcentration of seawater. The prokaryotic community presented some peculiarities, such as the occurrence of sequences of Patescibacteria (which can thrive in nutrient-limited water environments) or few Spirochaeta, and the presence of archaeal sequences of Methanomicrobia closely related to *Methanoculleus,* a methanogen commonly detected in marine and estuarine environments. The high percentage (35%) of unassigned fungal taxa suggested the presence of a high degree of undiscovered diversity within a structured fungal community (including both yeast and filamentous life forms) and reinforce the hypothesis of a high degree of biological uniqueness of the habitat under study.

## Introduction

Interest in brines within cryoenvironments has increased after they have been found on Mars (e.g.^1,2^). Brines were also discovered in the deep subsurface in Canada, Finland, Germany and Sweden. In Antarctica, hypersaline brines were found at McMurdo Sound, within the permafrost of the Taylor Valley (e.g.^3^), as well as below ice-sealed Antarctic lakes (e.g.^4^) or in the Antarctic subglacial lakes (i.e.^5^). Several studies modeled the occurrence and the effects of the subglacial brines or detected their occurrence through indirect methods (e.g.^3,6–8^). Despite these efforts the subglacial aquatic systems remain poorly understood mainly due to the lack of direct sampling. Englacial brines that flow within glaciers^9^, ice sheets or ice shelves^10^ are even less known. The connections between englacial and subglacial brines and their origins are still debated although at least in Taylor Valley several studies (i.e.^3^) provided strong inputs to their comprehension. Different mechanisms regarding the origin of the hypersaline brines were proposed (e.g.^9,11^). Subglacial and endoglacial brines can be extremely important in regard to glacier dynamics (e.g.^12,13^) and even more for ecological interest (e.g.^14^). Microbial communities sharing subglacial environment have been studied in recent decades, especially in order to understand possible effects on the weathering of the underlying rock (e.g.^15–17^). Probably, the most studied case is the outflow brine of Blood Falls in Taylor Valleys (e.g.^18–20^) that revealed the presence of a thriving community of chemosynthetic bacteria whose 74% of clones and isolates shared high 16S rRNA gene sequence homology with phylotypes from marine systems.

The microbial communities of hypersaline brines observed below ice-sealed Antarctic lakes were surely more investigated (i.e.^21–29^); accordingly, they have been used as comparison in this paper. The phylogenetic groups found in the different frozen lakes were quite different each other and exhibited different dominant groups: e.g. Bacteroidetes and Actinobacteria in East Bonney Lake or Proteobacteria (and Cyanobacteria^26^) in Lake Vanda^27^.

**Figure 1:**
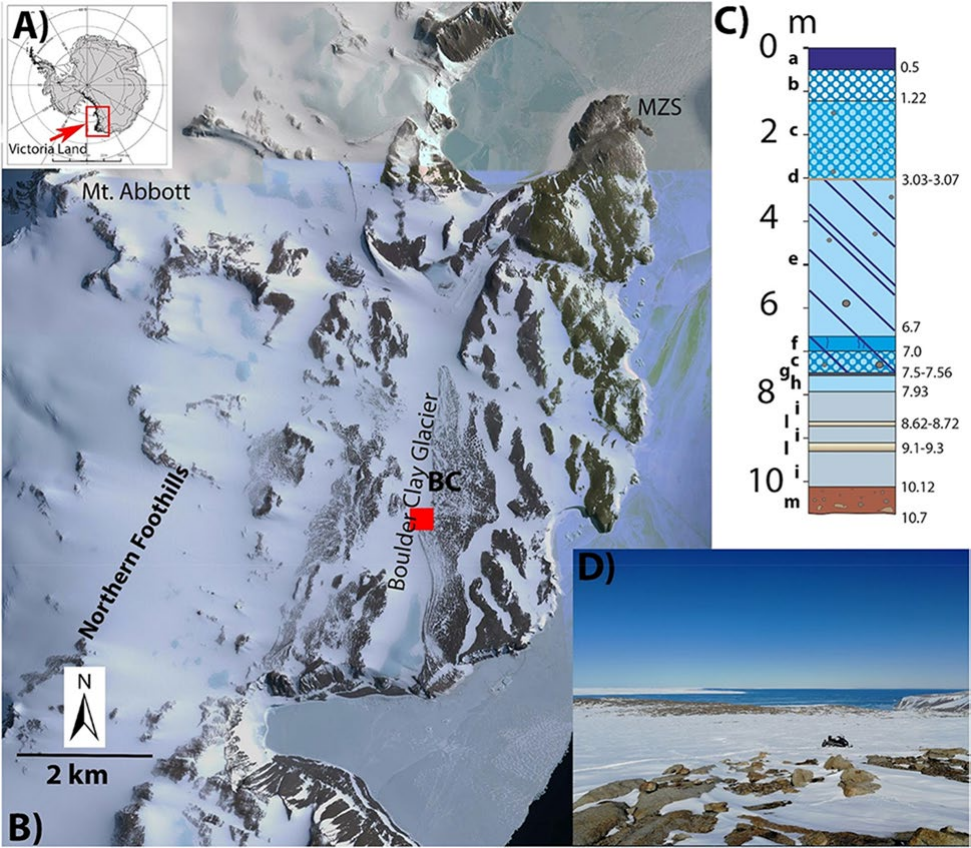
(**A**) Location map of the study area. (**B**) georeferenced satellite image with the location of the borehole (BC), the Italian Antarctic Station Mario Zucchelli (MZS) and some localities cited in the text. (**C**) Stratigraphy of the core (a, snow and firn; b) milky bubbly ice; c) bubbly ice with few clasts; d) well sorted frozen sand layer; e) clean ice with few clasts; f) ice with vertical tubules; g) ice with vertical tubules and black inclusions; h) clean ice; i) salty ice; l) salty slush (8.62–8.72) and brine (9.1–9.3); m) frozen till. (**D**) View of the borehole location from W to E.

Here we described the biotic and abiotic unicity of a hypersaline flowing endoglacial brine sampled through a borehole cored during the austral summer 2019 on the Boulder Clay Glacier, a coastal cold based glacier not far from the Italian Antarctic station (Mario Zucchelli Station, MZS, 74° S, Fig. 1). This study enhances the importance and the unicity of an endoglacial ecosystem that differs from all the others known to date, and opens a provocative question on the origin of the brine and its related ecosystem.

## Results

### The endoglacial hypersaline brine system

**Figure 2:**
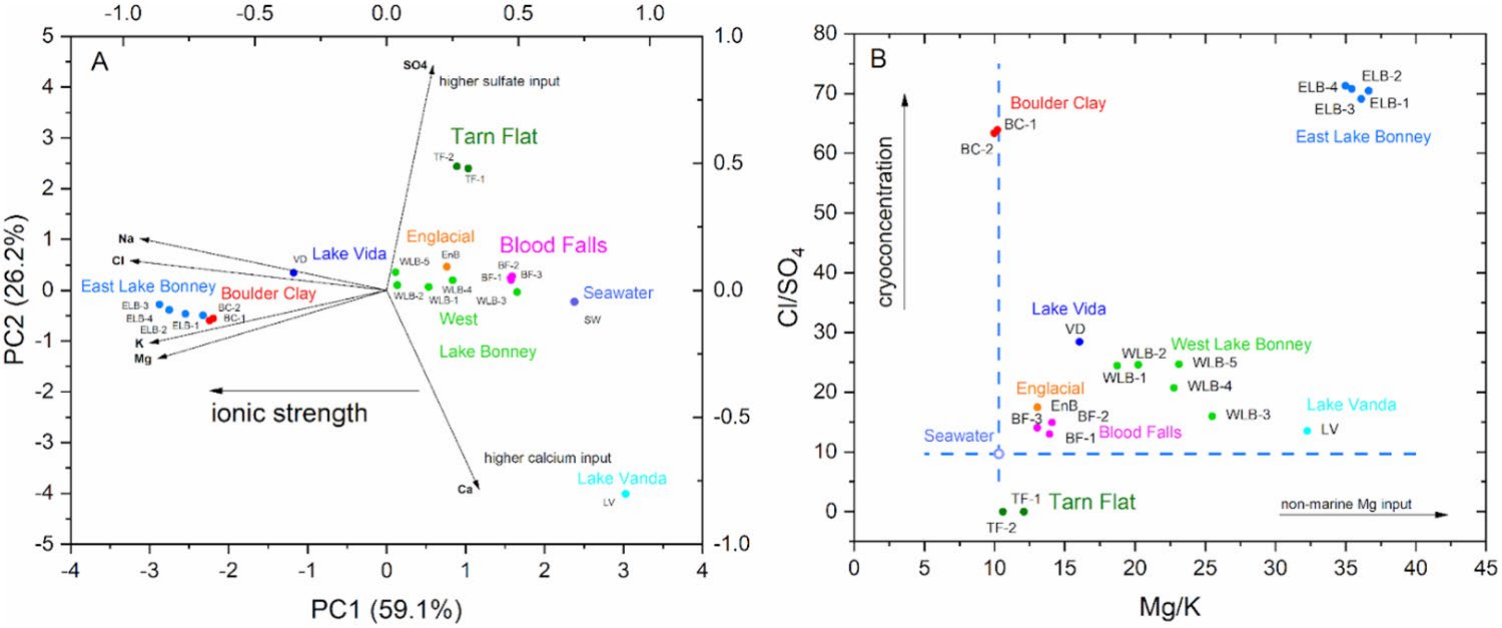
(**A**) Biplot PC1 vs PC2 (**B**) Cl/SO_4_ vs Mg/K.

The brines were found at 9.1 m of depth within a valley glacier characterized by a glacial unconformity and an erosional channel at 3 m of depth in which thin alluvial sediments (3 cm thick) were found. These peculiar structures are described in Forte et al.^30^. The ice below the unconformity appears almost similar down to 7.9 m of depth where it became slightly salty until 8.62 m where started a layer of salty, yellowish slush that at 9.1 m of depth became completely liquid (brine) at the date of sampling (26 November 2019) and remained liquid and flowing also at the last check (7 December 2019) when the level of brines raised upward 2.25 m. In situ the brine appeared quite rich in gas bubbles, with a temperature of − 17.4 °C, slightly basic (from 7.4 and 7.6), hypersaline (NaCl concentration is about 198 g L^−1^) and with a low percentage of DO (12.9%). The geochemical characteristics of the two samples of the same brine (BC-1 and BC-2) are reported in Table S1. The chemical analysis showed high contents in chlorides (3.8 eq L^−1^), sodium (2.7 eq L^−1^), magnesium (1.1 eq L^−1^) and potassium (110 meq L^−1^) with values that are close to only a few other Antarctic brines, such as the East Lake Bonney (ELB)^31^ and lake Vida brines (VD)^21^ although BC brines slightly exceed in sodium and potassium with respect to ELB and Lake Vanda (LV). Differently, sulfates (60 meq L^−1^) and calcium (160–170 meq L^−1^) showed values that are in line with the majority of the other Antarctic brines (e.g.^9,21,22,31^). The chemical peculiarity of BC brines with respect to the other Antarctic ones was preliminarily evaluated though Principal Component Analysis (PCA), including the six major ion concentrations as variables (see Tables S2–S4 for further details). Figure 2A shows the biplot of the first two PCs that explain together the 85% of the total variance. The PC1 (59%) is characterized by negative loadings of Mg^2+^, Na^+^, K^+^ and Cl^−^ (see Table S3), while PC2 (26%) has positive/negative loadings of SO_4_/Ca (0.886/− 0.783, respectively). PC1 significantly correlates with the ionic strength of the brines (see Table S1) with a r-Pearson correlation coefficient of − 0.91 (*p* value < 10^–6^). Therefore, PC1 can be interpreted as indicative of the ionic activity of the brine solution. ELB, BC and, to a lesser extent, VD brines have the more negative PC1 values. The PC2, instead, indicates the main characteristics of the less saline LV and Tarn Flat (TF) brines, characterized by a more significant input of Ca^2+^ and sulfates, respectively. Figure 2B shows the Mg/K ratio vs Cl/SO_4_ of several Antarctic brines, where it is possible to observe that Cl/SO_4_ of BC brines is approximately 10 times higher than seawater (SW), but similar to ELB brines. Conversely, Mg/K ratio in BC is almost the same as the seawater and it is sensibly lower than the Englacial and Blood Falls brines that were the most similar to BC for this parameter.

Another peculiarity of BC brines (see Table S1) is the total N content of about 42–43 mM, consistently higher than other brines. It must be noted that we did not detect any significant signal due to NO_2_ or NH_4_^+^, while the overall contribution of inorganic nitrogen was due to NO_3_. Similarly, to what was found by Lyons et al.^9^, we suppose that this value is an oxidation artifact due to the storage before the analysis. Moreover, the determination of the total N with the independent analysis carried out with Elemental Analyzer, provided values similar to NO_3_. Therefore, we prefer to report the total N concentration without any chemical speciation.

## The microbial diversity

### Prokaryotic diversity

Analysis of amplicon sequencing variants (ASVs) revealed a total number of 1042 ASVs, which were then resolved in 25 prokaryotic phyla, with a resolution of 162 genera. Unclassified sequences were on average 6.9 and 12.9% at phylum and genus levels, respectively.

Archaea were present in a low percentage (0.5%) almost entirely affiliated to the genus *Methanoculleus* (87%). Eight bacterial phyla occurred at a percentage greater than 0.1%, namely Actinobacteria, Bacteroidetes, Cyanobacteria, Firmicutes, Patescibacteria, Planctomycetes, Proteobacteria and Verrucomicrobia. Among them, Proteobacteria, Bacteroidetes, Verrucomicrobia, Patescibacteria, and Actinobacteria exceeded 1%. In particular, Proteobacteria (average percentage 48.6%) were particularly abundant and mainly represented by Gammaproteobacteria (family Marinobacteraceae) and, to a lesser extent, Alphaproteobacteria. The second most abundant group was that of Bacteroidetes (27.6%; mainly Flavobacteriaceae), followed by Verrucomicrobia, Patescibacteria and Actinobacteria (7.1, 4.5 and 3.2%, respectively). Firmicutes, Planctomycetes and Cyanobacteria were in the range 0.3–0.7%.

**Figure 3:**
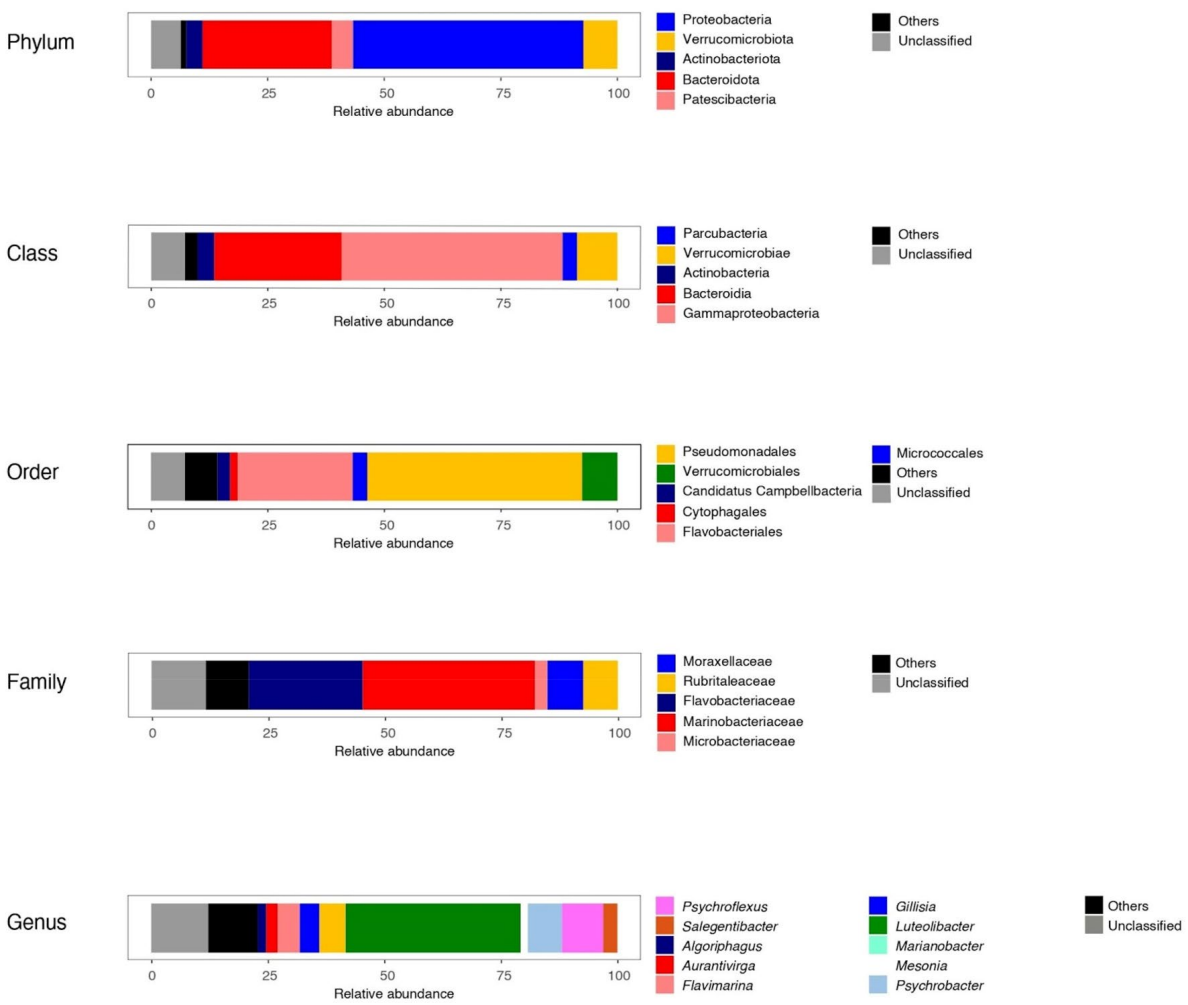
Bacterial community composition of BC brine at different classification levels. Only relative abundances greater than 1% are reported.

At genus level, *Marinobacter* (among Proteobacteria) was predominant (38.5%), followed at a lesser extent by *Psychroflexus* and *Flavimarina* (8.3 and 5.1%, respectively; among Bacteroidetes), *Psychrobacter* (7.8%; among Proteobacteria), and *Luteolibacter* (5.5%; among Verrucomicrobia). Other genera occurring at a percentage between 1 and 5% were *Gillisia*, *Salegentibacter*, *Aurantivirga*, *Mesonia* and *Algoriphagus*, all among Bacteroidetes (63.8% of Bacteroidetes sequences were resolved at genus level). Ten genera were found among Actinobacteria, but none of them exceeded 1%. No genus was determined among Patescibacteria (Fig. 3).

### Fungal diversity

**Figure 4:**
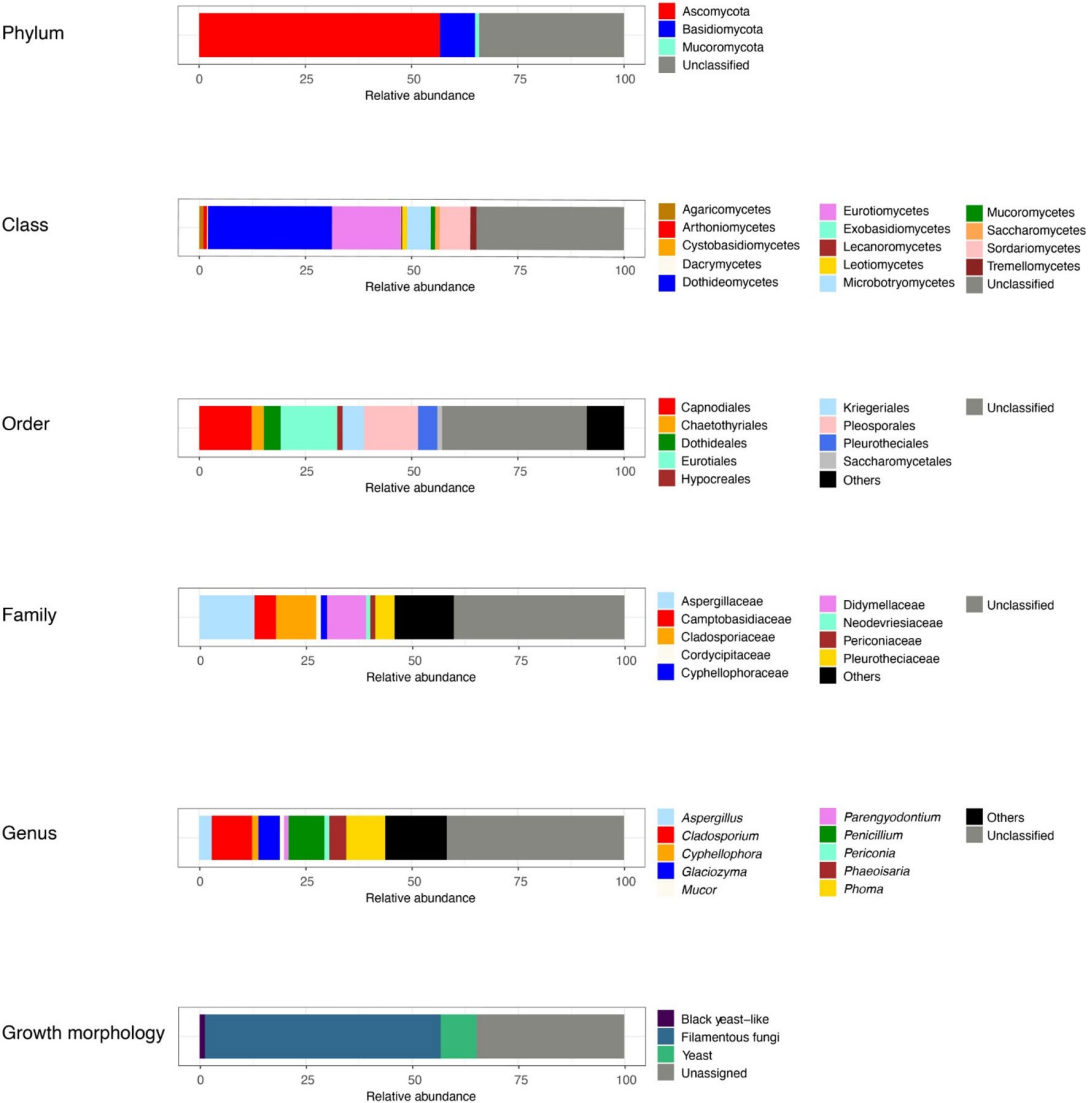
Fungal community composition of BC brine at different classification levels. Only relative abundances greater than 1% are reported.

A total of 141 ASVs belonging to three different fungal phyla were found. A high percentage (> 25%) of ASVs was assigned to unclassified fungi. At phylum level, 56.71% of ASVs were represented by Ascomycota, followed by Basidiomycota with 8.2%, and Mucoromycota with 1%. At genus level, the most represented taxa were *Cladosporium* (10%), *Phoma* (9%), *Penicillium* (8%), *Phaeoisaria* (4%), *Aspergillus* (3%), and to a lesser extent *Cyphellophora (*1.5%*)*, *Periconia* (1.2%), *Parengyodontium (*1%), and *Mucor (1%).* Considering yeast life forms, *Glaciozyma* was the unique yeast genus exhibiting an abundance > 1%. In addition, several rare genera (< 1%) were grouped as others (14%). The analysis of the fungal growth morphology showed that the brine was characterized mainly by filamentous fungi life forms (55.56%), followed by yeasts (8.44%) and black yeast − like fungi (1.23%) (Fig. 4).

## Discussion

### Endoglacial brines as a unique ecosystem for microbial life

To date, researchers have mainly focused their attention on prokaryotic diversity of Antarctic brines^18,23–25^, while fungal diversity has not yet been explored extensively^22,26^. Regarding the endoglacial brines of Vida, Vanda and Bonney (west and east lobes) lakes, a number of papers have been published since the last 1990s on the biotic and abiotic characteristics e.g.^21,27–29^. Even in this case, however, the study of microbial communities was almost exclusively aimed at the analysis of the prokaryotic fraction.

The occurrence of halophiles has been documented in worldwide hypersaline lakes: most of them belong to the phyla Proteobacteria, Cyanobacteria, Rhodothermaeota, Firmicutes, Actinobacteria, Bacteroidetes and Spirochaetes^32^. However, phylogenetically the prokaryotic assemblage of BC brine showed both similarities and divergences with other Antarctic hypersaline systems. A number of phylogenetic groups (such as Cyanobacteria and Planctomycetes) that are widespread in freshwater and marine ecosystems were observed in BC brines, as well as in East Bonney and Vanda Lakes, but they were not detected in Lake Vida. Differently from East Bonney Lake, where Bacteroidetes and Actinobacteria were predominant^26^, Proteobacteria dominated within the bacterial communities of BC brines. The dominance of Proteobacteria (and Cyanobacteria) was also reported by Ramoneda et al.^33^ in Lake Vanda. However, this result referred to microbial mats, whereas water under the ice cover of the lake was characterized by a high relative abundance of Actinobacteria. In the East Bonnie Lake, Proteobacteria were the next most abundant phylum after Bacteroidetes and Actinobacteria, and mainly consisted of Betaproteobacteria, which were instead absent in BC brines.

Gammaproteobacteria also occurred in the hypersaline East Bonney Lake, becoming dominant only in its deeper layers^29^, and in Lake Vanda^34^, thus highlighting their adaptation to high salinity levels. Consistently with Lake Vanda, the proteobacterial fraction of BC brines prokaryotic community included Alpha- and Gammaproteobacteria, whereas Deltaproteobacteria were not detected. The prokaryotic assemblage of BC brines also distinguished from those of Lake Vida and Blood Falls, as well as from East Bonney and Vanda Lakes, because it contains Patescibacteria as an exclusive phylotype. Patescibacteria (as well as Verrucomicrobia and Actinobacteria) are generally reported as bacterial inhabitants of permafrost (e.g.^35–37^). Interestingly, members of Patescibacteria can thrive in nutrient-limited water environments having simplified genomes which drive functions essential to growth and reproduction and retain stress response systems^38^. Further, the prokaryotic assemblage of BC brines (as observed for Lake Vida) harbored few Spirochaeta-related sequences unlike those of the Blood Falls brine and East Bonney Lake. These microorganisms and their metabolic features (such as H_2_ utilization, EPS production and fermentation) may be useful to describe the functional ecology of briny systems.

Another noteworthy feature of the BC brines concerned the occurrence, albeit scarce, of Archaea (not detected, for example, in Lake Vida). Significant diverse archaeal populations were reported for cold briny habitats of marine origin, such as the Vestfold Hills lake system in Eastern Antarctica^39^, and Lake Vanda^34^. Differently from observations on Lake Vanda, sequences of Methanomicrobia detected in BC brines were most closely related to *Methanoculleus* (instead of the methylotrophic *Methanomassiliicoccus*^34^)*,* a genus of methanogens commonly detected in marine and estuarine environments (e.g. shallow sediments^40^), but rarely reported for lacustrine habitats (e.g.^41^). These hydrogenotrophic methanogens well adapt to low H_2_ concentration, therefore possibly having an advantage over other methanogens in saline environments. Based on this result, *Methanoculleus* representatives are the most likely active methanogens in BC brines. Thus, differently from Lake Vanda brine, methanogenesis in BC brines occurs by the most widespread pathway, i.e. hydrogenotrophic methanogenesis, which has been suggested to be the ancestral form of methane production^42^.

At genus level, the gammaproteobacterial *Marinobacter* and *Psychrobacter* lineages are cosmopolitan and ecologically relevant in icy brines (e.g.^20,21^). The predominance of sequences related to the *Marinobacter* genus makes the prokaryotic assemblage of BC brines highly similar to those reported for brines of the Lake Vida and Blood Falls^21^. *Marinobacter* members were also abundant at 30 m-depth of the hypersaline East Bonney Lake^29^. *Marinobacter* spp. are aerobic with a strictly respiratory type of metabolism. They can grow anaerobically by denitrification coupled to the oxidation of a suitable donor carbon substrate and Na^+^ is required for growth. Among Bacteroidetes, the genus *Psychroflexus* is found within moderately hypersaline ecosystems across the world, including sea-ice^43^, salt lake (e.g.^44,45^), salt pan^46^ and marine solar saltern^47^. Members of this genus are generally aerobic, slightly or moderately halophilic.

Considering fungal communities, despite the presence of halophilic fungi in hypersaline systems were documented^32^, the almost total absence of analyses aimed at studying the eukaryotic fraction makes difficult (or even impossible) any comparison with the other endoglacial brines found in the Vida, Vanda and East Bonney lakes. The sole comparison that can be made is that with the study of Murray et al.^21^, who reported that PCR surveys to detect both eukaryal and archaeal SSU rRNA genes from genomic DNA were negative, thus assuming the absence of eukaryotic microbial populations (including both yeast and fungal life forms) in endoglacial brines of Vida lake. This result underlines the strong difference between them and the BC brines, where the presence of a structured fungal community was found.

In detail, the percentage of filamentous fungi found was higher (55.56%) than other growth morphology (i.e. yeasts and black yeast-like fungi), differently from the brine sampled in a frozen lake of Tarn Flat where yeasts dominated the fungal diversity^22^, and in another frozen lake of Boulder Clay, where the percentage of filamentous fungi and yeast form was similar^26^. Moreover, the high percentage of unassigned taxa (35%) found in the BC brines suggests the presence of high undiscovered fungal diversity, reinforcing the hypothesis of a high degree of biological uniqueness.

Although some of them can be found in extreme habitats worldwide^48,49^, the most abundant filamentous fungal genera colonizing the BC brines (*Aspergillus*, *Cladosporium* and *Penicillium*), as well as some minor genera (*Engyodontium*, *Phoma*), have been currently found in Antarctic seawaters and marine-associated habitats (e.g.^50,51^). Likewise, *Glaciozyma*, which is the unique yeast genus exhibiting an abundance ≥ 1%, has been widely recovered in seawaters and marine-associated habitats^50,51^. It was also found in Antarctic brines from Tarn Flat^22^. On the basis of these results, and in analogy to what is reported above for bacterial taxa, the hypothesis of possible marine origin of the BC brines cannot be excluded.

Black yeast-like fungi exhibit morphological and physiological characteristics making them the organisms well adapted to the harsh Antarctic conditions. This microbial group is characterized by wide environmental plasticity and their ability to shift from one growth form to another, according to the physicochemical environmental conditions, may be regarded as an adaptive strategy to stressful conditions (e.g.^52,53^). Their lower abundance in BC brines compared to filamentous fungi and yeasts is compatible with their lower growth rates, as reported by Canini et al.^54^. However, the presence of some melanized fungi (i.e. members of the genera *Aureobasidium*, *Hortaea*, *Phaeotheca* and *Trimmatostroma*) have been found in hypersaline environments worldwide^32^.

### BC endoglacial brines: a provocative question about their origin?

It is now widely accepted that hypersaline surface or subsurface waters (brines) have been pervasive on Mars, at least periodically, throughout the last 3.5 billion years, and may be present still today^1,55–58^.

Conversely hypersaline waters are not so common on Earth. However, in Antarctica that is considered the best Mars analogue for many reasons, these hypersaline waters are likely more widespread, at least in the Dry Valleys.

The hypersalinity of Antarctic brines has been explained through different mechanisms, although there is consensus that coastal Antarctic brines mainly come from the cryoconcentration of seawater^59,60^. Therefore, their chemistry is only scarcely influenced by rock-weathering and/or biogeochemical cycles contributions^61^ and the possible input deriving from the dissolution of halites is poorly convincing in East Antarctica^62^. The cryoconcentration of seawater provides the preferential precipitation of the less soluble sodium sulfate decahydrates (mirabilite) at temperature lower than − 8.2 °C. This mechanism is considered responsible for the depletion of Na^+^ and SO_4_^2−^. However, as observed by Cragin et al.^59^, the Na/SO_4_ ratio in brines is lower than in seawater, mainly due to the major relative changes in SO_4_^2−^ with respect to Na^+^, while Na_2_SO_4_ precipitates. In accordance with Cragin et al.^59^ observations, BC brines show a Na/SO_4_ ratio (meq/meq) of 0.022 that is about 5-times lower than seawater (i.e. 0.12). The effect of the cryoconcentration is also evident from the Cl/SO_4_ values reported in Fig. 2B, where the higher Cl/SO_4_ found in BC and ELB are clearly indicative of an enhanced cryoconcentration process. In addition, Mg/K ratios, here proposed as indicators of input from non-marine sources, such as weathering, rock-water interactions as well as biogeochemical cycles, shows BC values very close to seawater composition, while ELB provides values significantly higher. Therefore, the hypersalinity of BC brines is mainly originated by seawater after an intensive cryoconcentration that likely began to occur already in remote epochs. The other peculiarity of the BC brines is the high N content that together with the total P, resulted significantly higher than other brines. This occurrence suggests a prolonged or more intense biological activity in BC brines with respect to the other ones considered in this study. Considering the advanced seawater cryoconcentration and the very scarce contribution of rock weathering in conjunction with the probable more prolonged and/or more intense biological activity, the question about where and when these brines were generated arises. At this stage, we cannot provide a comprehensive answer, but we can attempt to formulate the following innovative hypothesis. Indeed, Lyons et al.^9^ have already explained that the end-member brine found below the Taylor glacier (Blood Falls), was derived from seawater and they speculated on the mechanism controlling cryoconcentration that led to the formation of the hypersaline water.

Differently, in our case the actual landscape does not show any evidence of ancient lakes or fjords or inland sea where seawater could be present in the remote past. In addition, we have to consider that according to Armienti and Baroni^63^, the glaciers of this sector of Victoria Land have preserved typical polar geomorphological features with negligible erosional power, during the last 8.2–7.5 millions of years. Apparently only the higher peaks of the Northern Foothills as the close Mt. Abbott (Fig. 1) were deglaciated since the mid Pliocene (3.85 Ma; Di Nicola et al.^64^) while, since that time, areas below 720 m asl have been repeatedly exposed and overridden by expanding ice bodies. On the other hand, if we consider Levy et al.^65^ at the end of the mid Pliocene, after the peak of the Pliocene warmth, when global average temperatures increased to 4 °C warmer than pre-industrial levels, a phase of tectonic uplift occurred. Consequently, the areas containing seawater and marine sediments (lagoon conditions?), where brines could have been formed, rose up. Although it is not possible to exclude that the origin of the brine can predate the Miocene, where, in some depressions, brines might had been preserved by the glacial erosion before the 8.2 Ma, even during the Early and Late Oligocene, when the Victoria Land coast was characterized by the presence of vegetation, such as *Nothofagus* spp., podocarps and bryophytes^66^.

## Methods

Boulder Clay Glacier is located (BC, Fig. 1) just a few kilometers from the Mario Zucchelli Italian Station (MZS). The glacier reaches the sea at Adelie Cove and eastward is limited by a debris-covered glacier that flows towards SSE. The borehole BC (red star in Fig. 1b) reached the brines at 9.1 m of depth. The borehole stratigraphy is represented in Fig. 1c. The measurements in situ were carried out with a multiparametric probe (Hanna Instruments—HI98194 model) a few minutes after the coring. Two BC brines (BC-1 and BC-2) were sampled with a peristaltic pump using sterile tubes in the same day at only a couple of minutes of distance. The samples were immediately transported to the labs of MZS and preserved at − 20 °C and successively in Italy at Messina Labs keeping the − 20 °C temperature during all the transport.

The chemical analyses of BC brines were carried out in the laboratory of Venice. All liquid brine samples were filtered using a PTFE membrane (pore size 0.45 µm) before analyses. The anions (NO_3_^−^, SO_4_^2−^ and Cl^−^) and cations (NH_4_^+^, Na^+^, K^+^, Mg^2+^ and Ca^2+^) were analyzed using ion chromatography (Metrohm 761 Compact IC Chromatography) equipped with a Cation 1–2 (particle size 7 μm; eluent: HNO_3_ 3 mM) and a Metrosep Anion supp/4 (particle size 5 μm; eluent: HCO_3_^−^/CO_3_^2−^ buffer 1.7/1.8 mM) column for cation and anion analysis, respectively. The brines were appropriately diluted in ultra-pure water to fit the calibration range and analyzed by ICP-MS using an iCAP RQ (Thermo Scientific) instrument equipped with an ASX-560 autosampler (Teledyne Cetac Technologies), PFA cyclonic spray chamber at 2.7 °C, sapphire injector, quartz torch, Ni cones and 1550 W of plasma radio frequency power. The ICP analysis was performed for Na, K, Ca, Mg and P. The Na, K, Mg and Ca values obtained with ICP-MS were not significantly different from those obtained with cationic IC and therefore we reported a mean value of the results obtained with these two techniques for these elements.

Total carbon and nitrogen were determined with a Flash 2000 HT Elemental Analyzer (Thermo Scientific). The principal component analysis was carried out with R Studio, where the variables (Na, K, Mg, Ca, SO_4_ and Cl) were preliminarily scaled.

### DNA extraction and NGS sequencing

For microbiological analyses, collected BC brines were pooled in a single sample and filtered (between 300 and 350 ml) in five replicates on polycarbonate filters (size 45 mm; porosity 0.22 µm). DNA was extracted from membranes using the Power Soil DNA extraction kit (MoBio Laboratories, Carlsbad, CA, USA) according to the manufacturer’s instructions. DNA concentrations and purity were quantified by using a NanoDrop ND-1000 UV–Vis spectrophotometer (NanoDrop Technologies, USA). Bacterial 16S rDNA region (V3–V4) and fungal internal transcribed spacer region 2 (ITS2) were amplified using the following primers:

i) IlluAdp_16S_341f 5′-CCTACGGGNGGCWGCAG-3′ and IlluAdp_16S_805r 5′-GGACTACHVGGGTATCTAATCC-3′ for bacterial 16S rRNA gene region V3–V4.
ii) IlluAdp_ITS31_NeXTf 5′-CATCGATGAAGAACGCAG-3′ and IlluAdp_ITS4_NeXTr5′-TCCTCCGCTTATTGATATGC-3′^67^ for fungi.

Sequencing was performed using the Illumina MiSeq platforms, following the standard protocols of the company IGA Technology Services Srl (Udine, Italy).

### Bioinformatics analysis

FastQC was used to check the quality of raw sequences^68^. Sequences were pre-processed, quality filtered, trimmed, de-noised, merged, modeled, and analyzed via DADA2 within QIIME2^69^ and chimeras were removed following the ‘consensus’ method reported by Callahan et al.^70^.

Bacterial taxonomy annotation was performed using Silva 138 99% ASVs full-length sequences (silva-138-99-nb-classifier.qza). Fungal taxonomy annotation was performed using a Naïve–Bayes classifier trained on the UNITE + INSD database against the representative sequences^71^. All sequences have been submitted to the National Center for Biotechnology Information (NCBI) under the BioProject PRJNA826749, with the biosample accession numbers SAMN27582456, SAMN27582457, SAMN27582458, SAMN27582459 and SAMN27582460 for bacteria, and SAMN27584063, SAMN27584064, SAMN27584065, SAMN27584066 and SAMN27584067 for fungi.

## Supplementary Information

Supplementary Information.

## Data Availability

The datasets used and/or analysed during the current study available from the corresponding author on reasonable request.
