# Supplementary material for: A possible unique ecosystem in the endoglacial hypersaline brines in Antarctica
Source: Sci Rep. 2023 Jan 5;13:177. doi: 10.1038/s41598-022-27219-2 (PMC9814585; doi:10.1038/s41598-022-27219-2)
Supplement: Supplementary file 1 — Supplementary Information. [file 41598_2022_27219_MOESM1_ESM.pdf]

Table S1. Geochemical characteristics of Boulder Clay Brines and similar environments.

Site	Ref.	ID	pH	Conductivity	Cl^-	SO_4^{2-}	Na^+	K^+	Ca^{2+}	Mg^{2+}	Ionic strength*	C_{tot}	N_{tot}	P_{tot}
				(mS cm^{-1})	(meq L^{-1})	(meq L^{-1})	(meq L^{-1})	(meq L^{-1})	(meq L^{-1})	(meq L^{-1})	(M)	(mM)	(mM)	(µM)
BC	This study	BC-1	7.2±0.1	172±3	3800±300	60±7	2700±110	110±10	160±30	1100±15	4.6	28±1	43±1	14±2
BC	This study	BC-2	7.2±0.1	176±4	3800±230	60±8	2700±110	110±10	170±25	1100±10	4.7	27±1	42±1	21±2
EnB	[1]	EnB	-	-	2043	117	1683	31	157	398	2.5	108[a]	0.009[b]	1.3[c]
BF	[1]	BF-1	-	-	1320	102	1117	19	109	270	1.7	-	-	-
“	[1]	BF-2	-	-	1450	97	1104	18	104	254	1.7	-	0.17[b]	0.7[c]
“	[1]	BF-3	-	-	1347	96	1106	20	105	258	1.7	55.4[a]	0.09[b]	-
WLB	[1]	WLB-1	-	-	2172	88	1535	34	108	688	2.8	78.8[a]	0.30[b]	0.3[c]
“	[2]	WLB-2	-	-	2299	94	1789	43	124	804	3.1	-	-	-
“	[3]	WLB-3	-	-	1246	78	970	15	79	392	1.7	-	-	-
“	[3]	WLB-4	-	-	1942	94	1474	26	99	587	2.5	-	-	-
“	[3]	WLB-5	-	-	2529	103	1930	34	111	775	3.2	-	-	-
ELB	[4]	ELB-1	-	-	4635	66	2140	70	69	2556	6.1	-	-	-

“	[4]	ELB-2	-	-	4798	67	2265	74	72	2588	6.3	-	-	-
“	[4]	ELB-3	-	-	5133	74	2553	73	68	2639	6.7	-	-	-
“	[4]	ELB-4	-	-	4949	70	2441	74	70	2634	6.5	-	-	-
LV	[5]	LV	-	-	217	16	294	20	1210	632	2.1	-	-	-
VD	[6]	VD	6.2	188 (psu)	3318	117	1914	83	60	1330	4.2	109[a]	4.8[b]	5[d]
TF	[7]	TF-1	-	-	< lod	221	< lod	14	40	167	0.4	58.8	-	-
“	[7]	TF-2	-	-	< lod	227	< lod	22	38	230	0.5	64.5	-	-
	[8]	SW	-	-	559	58	481	11	15	108	0.7	-	-	-

[a] total C given as the individual sum of HCO_3^-+dissolved organic carbon (DOC)+dissolved inorganic carbon (DIC). [b] total N given as the individual sum of NH_4^++NO_2^-+NO_3^-. [c] given as soluble reactive phosphate. [d] given as P-PO_4. [1] Lyons et al. 2019 [2] Lyons et al. 2020 [3] Dowling and Jones 2014 [4] Doran 2014 [5] Cartwright and Harris 1981 [6] Murray et al. 2012 [7] Borruso et al. 2018 [8] Pilson 2013. * The ionic strength was calculated as where z_i is the charge and C_i the molar concentration of the i-th ion.

Table S2 Eigenvalues and explained variance from the Principal Component Analysis performed for the 6 major ion concentrations (Cl, SO_4, Na, K, Ca and Mg) and 20 objects

**Principal Component (PC)**	**Eigenvalue**	**Variance**
**PC1**	**3.55**	**59.1**
**PC2**	**1.57**	**26.2**
PC3	0.47	7.8
PC4	0.35	5.9

PC5	0.05	0.9
PC6	0.004	0.06

Table S3 Loadings from the Principal Component Analysis performed for the 6 major ion concentrations (Cl, SO_4, Na, K, Ca and Mg) and 20 objects

**Variable**	**PC1**	**PC2**	**PC3**	**PC4**	**PC5**	**PC6**
Ca	0.351	**-0.783**	0.511	-0.041	0.024	0.003
Mg	**-0.870**	-0.270	-0.052	-0.406	-0.040	-0.030
Na	**-0.937**	0.204	0.144	0.180	0.165	-0.018
K	**-0.900**	-0.209	0.084	0.344	-0.145	-0.005
Cl	**-0.975**	0.117	0.089	-0.161	0.008	0.048
SO_4	0.177	**0.886**	0.411	-0.099	-0.064	-0.010

Table S4 Scores from the Principal Component Analysis performed for the 6 major ion concentrations (Cl, SO_4, Na, K, Ca and Mg) and 20 objects

	**PC1**	**PC2**	**PC3**	**PC4**	**PC5**	**PC6**
BC-1	-2.19	-0.56	0.24	1.37	0.02	0.08
BC-2	-2.24	-0.60	0.30	1.43	0.02	0.04
EnB	0.76	0.46	0.23	0.22	0.26	-0.04

BF-1	1.59	0.27	-0.36	0.06	-0.03	-0.04
BF-2	1.57	0.24	-0.42	0.04	0.01	0.06
BF-3	1.58	0.20	-0.44	0.09	-0.02	0.00
WLB-1	0.53	0.06	-0.30	0.08	0.13	0.01
TF-1	1.03	2.40	1.14	-0.33	0.02	0.09
TF-2	0.89	2.43	1.23	-0.26	-0.18	0.01
LV	3.03	-4.01	1.48	-0.38	-0.06	0.01
WLB-2	0.14	0.10	-0.07	0.22	0.17	-0.14
WLB-3	1.65	-0.04	-0.81	-0.06	-0.01	0.00
WLB-4	0.84	0.20	-0.33	0.01	0.20	-0.03
WLB-5	0.11	0.35	0.02	0.07	0.46	-0.06
ELB-1	-2.32	-0.49	-0.31	-0.79	-0.13	0.02
ELB-2	-2.55	-0.47	-0.22	-0.72	-0.09	0.02
ELB-3	-2.87	-0.28	-0.04	-0.74	0.18	0.03
ELB-4	-2.75	-0.39	-0.13	-0.71	0.06	-0.01
VD	-1.18	0.34	0.25	0.34	-0.70	-0.13
SW	2.38	-0.23	-1.45	0.06	-0.30	0.07

Table S5 Major ion ratios (meq/meq)

Site	ID	Na/Cl	Mg/K	Ca/K	Cl/SO_4	Mg/Ca	Ca/Cl	Ca/SO_4	SO_4/Na

BC	BC-1	0.70	10.3	1.5	63.9	7.0	0.041	2.60	0.022
BC	BC-2	0.71	10.0	1.5	63.6	6.5	0.045	2.83	0.022
EnB	EnB	0.82	12.8	5.1	17.5	2.5	0.077	1.34	0.070
BF	BF-1	0.85	14.2	5.7	12.9	2.5	0.082	1.07	0.091
"	BF-2	0.76	14.1	5.8	14.9	2.4	0.072	1.07	0.088
"	BF-3	0.82	12.9	5.3	14.0	2.5	0.078	1.10	0.087
WLB	WLB-1	0.71	20.2	3.2	24.7	6.4	0.050	1.22	0.058
"	WLB-2	0.78	18.7	2.9	24.5	6.5	0.054	1.32	0.053
"	WLB-3	0.78	26.1	5.3	16.0	5.0	0.064	1.02	0.080
"	WLB-4	0.76	22.6	3.8	20.7	5.9	0.051	1.06	0.064
"	WLB-5	0.76	22.8	3.3	24.6	7.0	0.044	1.09	0.053
ELB	ELB-1	0.46	36.5	1.0	70.2	37.0	0.015	1.05	0.031
"	ELB-2	0.47	35.0	1.0	71.6	35.9	0.015	1.06	0.031
"	ELB-3	0.50	36.2	0.9	69.4	38.8	0.013	0.91	0.030
"	ELB-4	0.49	35.6	0.9	70.7	37.6	0.014	1.00	0.029
LV	LV	1.35	31.6	60.5	13.6	0.5	5.58	75.50	0.055
VD	Vida	0.58	16.0	0.7	28.4	22.2	0.018	0.52	0.061
TF	TF-1	-	11.9	2.9	-	4.2	-	0.18	-
"	TF-2	-	10.5	1.7	-	6.1	-	0.17	-
SW	SW	0.86	9.8	1.4	9.6	7.2	0.027	0.26	0.120

Table S6 Seawater Enrichments [meq/meq] of the major ions. Seawater chemical composition from Pilson 2013.

**Site**	**ID**	**Cl^-**	**SO_4^{2-}**	**Na^+**	**K^+**	**Ca^{2+}**	**Mg^{2+}**
BC	BC-1	6.9	1.0	5.6	10.2	10.4	10.1
BC	BC-2	6.8	1.0	5.7	10.5	11.4	10.5
EnB	EnB	3.7	2.0	3.5	2.9	7.3	2.5
BF	BF-1	2.4	1.8	2.3	1.8	7.3	2.5
“	BF-2	2.6	1.7	2.3	1.7	7.0	2.5
“	BF-3	2.4	1.7	2.3	1.9	7.1	2.3
WLB	WLB-1	3.9	1.5	3.2	3.2	7.3	2.4
“	WLB-2	4.1	1.6	3.7	4.1	8.3	7.4
“	WLB-3	2.2	1.3	2.0	1.5	5.3	3.6
“	WLB-4	3.5	1.6	3.1	2.5	6.7	5.4
“	WLB-5	4.5	1.8	4.0	3.2	7.5	7.2
ELB	ELB-1	8.3	1.1	4.5	6.6	4.6	23.6
“	ELB-2	8.6	1.2	4.7	7.0	4.8	23.9
“	ELB-3	9.2	1.3	5.3	7.0	4.5	24.4
“	ELB-4	8.9	1.2	5.1	7.1	4.7	24.3

LV	LV	0.4	0.3	0.6	1.9	81.1	5.8
VD	VD	5.9	2.0	4.0	7.9	4.0	12.3
TF	TF-1	-	3.8	-	1.3	2.7	1.5
“	TF-2	-	3.9	-	2.1	2.6	2.1
